# Frantz tumor with asymptomatic microscopic hematuria: Case report

**DOI:** 10.1097/MD.0000000000043958

**Published:** 2025-08-22

**Authors:** Haixia Ren, Chongxi Ren, Sheng Li

**Affiliations:** a Department of Gynecology and Operating room, Hebei Cangzhou Hospital of Integrated Traditional Chinese and Western Medicine, Cang Zhou, China; b Department of Surgical Oncology, Hebei Cangzhou Hospital of Integrated Traditional Chinese and Western Medicine, Cang Zhou, China; c Department of General Surgery, Hebei Cangzhou Hospital of Integrated Traditional Chinese Medicine and Western Medicine, Cang Zhou, China.

**Keywords:** asymptomatic microscopic hematuria, franz tumor, pancreatic tumor, solid pseudopapillary neoplasm, surgery

## Abstract

**Rationale:**

Franz tumor, which has been classified as solid pseudopapillary neoplasm (SPN) of the pancreas, is a rare tumor with low malignant potential that occurs mainly in young women. Its clinical manifestations are atypical or asymptomatic, diagnosis is often incidental or detected by imaging for other reasons, and little is known about their optimal treatment. This analysis aimed to increase knowledge about the occurrence and treatment strategies of SPN.

**Patient concerns:**

A 17-year-old female who presented for 6 weeks with asymptomatic microscopic hematuria (AMH) was admitted after an outpatient ultrasound examination revealed a pancreatic mass.

**Diagnoses:**

Abdominal enhanced computed tomography and magnetic resonance imaging revealed a solid pseudopapillary tumor of the pancreas, the large tumor involving the splenic artery and pressing on the left kidney.

**Interventions:**

The patient underwent distal pancreatectomy and splenectomy, and SPN was confirmed by histopathology.

**Outcomes:**

She did not experience any complications and was discharged from the hospital after recovery. Postoperative reexamination and the 4-month follow-up showed that the hematuria disappeared and the tumor did not recur.

**Lessons:**

The diagnosis of SPN is often incidental, as most patients are asymptomatic or have atypical symptoms. Most of the pathological manifestations were low-grade tumors. Surgical resection is still the standard of care. AMH caused by SPN is extremely rare, and the association may be attributed to exogenous tumor growth involving the kidney. As with getting an early diagnosis, surgery can be a challenge, but patients with SPN are associated with an excellent prognosis after surgery. Suspicion and proper investigation are essential to diagnose this entity in a timely manner.

## 1. Introduction

Frantz tumor, initially characterized by Dinarvand et al,^[1]^ has been classified as a low-grade solid pseudopapillary neoplasm (SPN) of the pancreas.^[2]^ It is a rare type of pancreatic tumor, accounting for only 2% of exocrine tumors of the pancreas.^[3]^ SPN is found throughout the pancreas, but more commonly in the body or tail,^[4]^ and it usually has no obvious clinical symptoms or some nonspecific manifestations. Approximately 70% of cases are asymptomatic or have only some nonspecific manifestations, the most common of which is vague abdominal pain. And the disease is often detected incidentally on imaging or examination.^[5]^ Due to its low-grade malignant potential, the lesion is usually clear, the standard treatment is surgical removal, and the patient has a good prognosis.

Despite an increase in reported cases over the past 20 years due to significant advances in clinical knowledge, imaging techniques, and immunohistochemical methods,^[6]^ no cases of SPN with asymptomatic microscopic hematuria (AMH) have been reported to date. Here, the present report details a 17-year-old girl who was diagnosed with SPN due to outpatient visits for AMH, and reviews the literature on this rare type of tumor. The case report has been reported in line with the SCARE 2023 criteria.^[7]^

## 2. Case presentation

A 17-year-old adolescent girl with AMH for 6 weeks was admitted to our hospital after ultrasound diagnosis of a pancreatic mass. The patient did not complain of lumbar and abdominal pain. No symptoms such as frequent urination, urgent urination, urination pain, gross hematuria and other conditions.There was no abdominal distension, fever, jaundice, nausea, vomiting, heartburn, weight loss, or loss of appetite.No abnormal menstrual history.The patient had no prior medical or surgical history and no history of alcohol or drug use. The patient had no obvious family history.

The patient is generally in good condition with vital signs within normal range. The abdomen was slightly distended, and gastrointestinal type and peristaltic wave were not observed. No scar or varicose veins; soft texture, palpable spherical mass on the left side of the upper abdomen, smooth surface, no tenderness, poor movement, unclear boundary, approximately 10 cm in diameter; Percussion showed drum sound, no mobile dullness; And normal bowel sounds heard on abdominal examination. Bilateral renal percussion pain was negative.

Urine chemical analysis and visible component analysis (urine test) showed red blood cell count (RBC) 33/μL, white blood cell count (WBC) 5/μL, occult blood (++). Blood and urine amylase were normal. Tumor markers, including alpha-fetoprotein (AFP), carbohydrate antigen (CA199), and carcinoembryonic antigen (CEA), were within normal ranges. Hemoglobin (HB) was 13.2 g/dl, RBC 4.32 × 10^12^/L, WBC 9.56 × 10^9^/L, platelet 170 × 10^9^/L, BUN 13 mg/ dl, Creatinine 0.51 mg/dl, Na 131 mmol/L, K 3.57 mmol/L, Lipase 17 U/L, GPT 13 U/L, CRP 0.312 mg/dl, CEA 0.71 ng/ml, CA199 3.87 U/ml, CA125 17.6 U/ml, AFP 1.2ng/ml.

**Figure 1.**
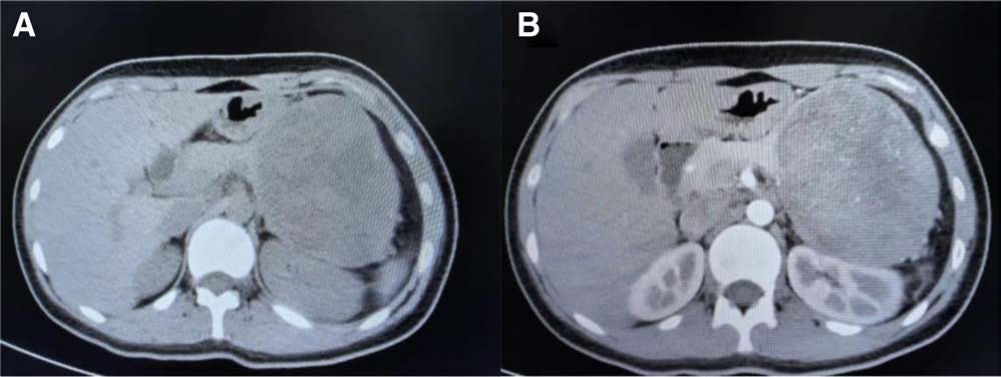
Abdomen CT scan with and without contrast showed a well-defined rounded hypodense non-enhancing parenchymal solid mass lesion in the body and tail of the pancreas, measuring approximately 10.5 cm × 8.2 cm × 10.4 cm. The mass involves the left kidney and compresses it backward and downward. (A) Plain CT scan; (B) contrast-enhanced CT. CT = computed tomography.

**Figure 2.**
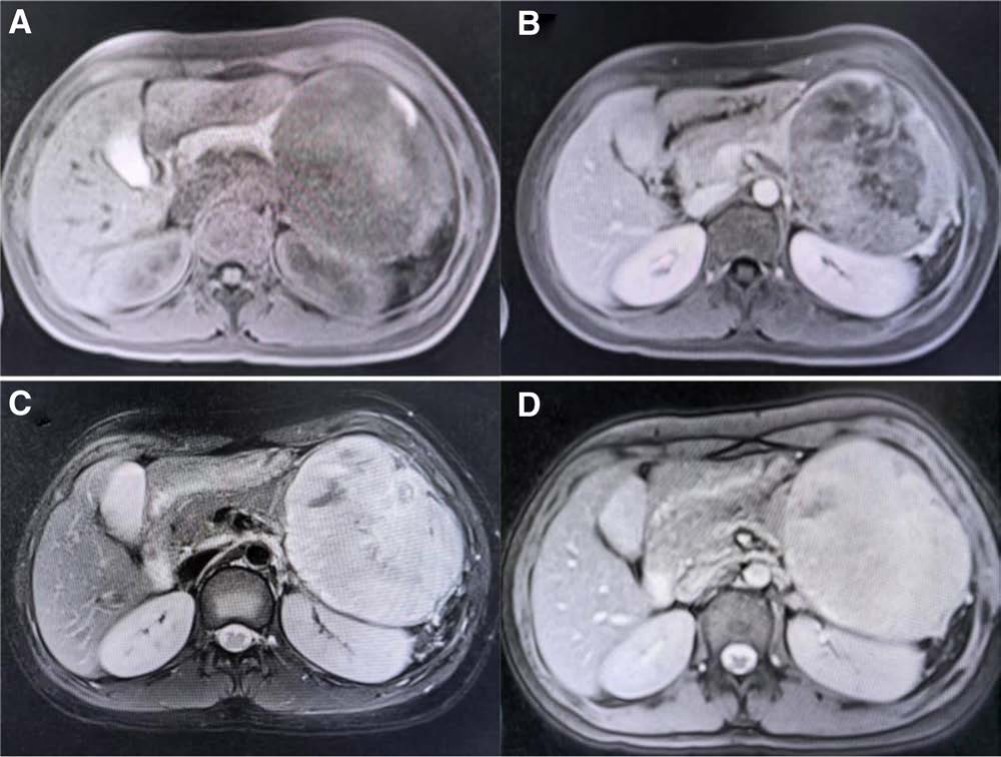
Abdominal MRI revealed large pancreatic mass and altered signal intensity. The mass involved the distal pancreas, pressing on the left kidney and forming a “concave line” on the renal side. (A) T1; (B) T1 + contrast; (C) T2; (D) T2 + contrast. MRI = magnetic resonance imaging.

Abdomen computed tomography (CT) scan with and without contrast showed a well-defined rounded hypodense non-enhancing parenchymal solid mass lesion in the body and tail of the pancreas, measuring approximately 10.5cm × 8.2cm × 10.4cm (Fig. 1). Abdominal magnetic resonance imaging (MRI) revealed large pancreatic mass and altered signal intensity. The mass involved the distal pancreas, pressing on the left kidney and forming a “concave line” on the renal side (Fig. 2).

Experienced surgeons performed complete tumor resection under general anesthesia in a supine position. Given the patient’s young age, we attempted a splenic-sparing distal pancreatectomy. However, a distal pancreatectomy and splenectomy were performed because splenic artery involvement made it difficult to preserve the spleen. The left superior rectus abdominis was incised into the abdomen without ascites, looking directly at the bulge at the gastrocolic ligament. The minor omental sac was opened, and the distal part of the pancreas was occupied by a spherical mass about 10 × 10 × 8 cm. The large mass presses down on the left kidney, forming a concave plane on the renal surface between them, with no fat layer and no obvious adhesion. After dissection, severing and ligation of the splenic artery at the upper margin of the pancreas, the superior mesenteric artery was dissected, exposed and protected, the pancreas was severed laterally, and the distal pancreas and spleen were removed. Lymph nodes in areas 10, 11 and 14 around the tumor were removed. Tumor resected in total was sent for histopathology. The abdomen was closed in layers, and the skin was approximated using staples.

The postoperative period went uneventful.

**Figure 3.**
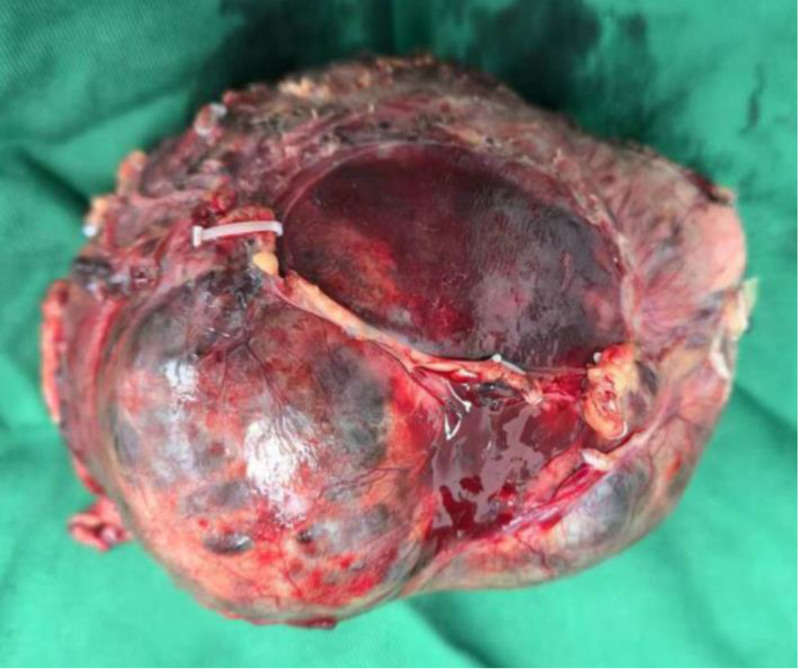
Gross appearance of the distal pancreatic mass specimen (SPN) showing well-defined lesion of approximately 12.5 cm × 10.0 cm × 7.8 cm. SPN = solid pseudopapillary neoplasm.

**Figure 4.**
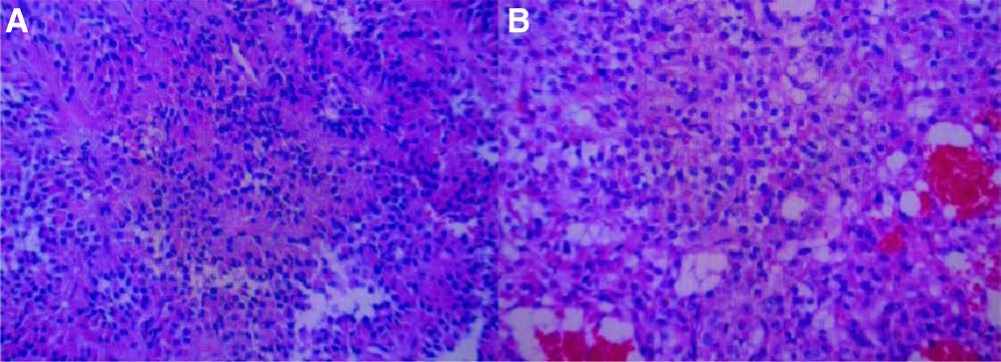
Histopathology confirmed solid pseudopapillary tumors of the pancreas (body and tail). Microscopic examination: (A and B) solid pseudopapillary neoplasms are composed of poorly cohesive epithelial cells forming solid and pseudopapillary structures. The pseudopapillae are formed by epithelial cells loosely arranged around hyalinised stroma that contains thin-walled blood vessels. The neoplastic cells are small and monomorphic, cytoplasm eosinophilic or transparent, and lack mucin. The nucleus is round or oval, without atypia. The chromatin structure of the nucleus is fine, and the nucleolus is absent or not obvious. (B) The tumor has hemorrhagic, necrotic and cystic changes. Focal cholesterol crystallization, hemosiderin deposition with calcification, and multinuclear giant cell reaction are seen.

Gross appearance of the distal pancreatic mass specimen (SPN) showing well-defined lesion of approximately 12.5 cm × 10.0 cm × 7.8 cm (Fig. 3). Histopathology confirmed solid pseudopapillary tumors of the pancreas (Fig. 4). There were 17 lymph nodes around the tumor, all of which showed chronic inflammation.

After the surgical intervention, the patient did not receive any additional chemotherapy, radiotherapy or other treatments. There was no fever after the surgery, no complications such as pancreatic leakage, abdominal infection, or lung infection occurred, the incision healed well, and the patient felt no discomfort. The patient was discharged from the hospital after recovery. She was followed up for 4 months with normal urine tests and renal function, and no tumor recurrence.

## 3. Discussion

SPN of the pancreas is rare in adolescent females. It is usually found incidentally during radiology or routine physical examination for other reasons, without typical clinical signs and symptoms. In the clinical case presented, the patient was asymptomatic, and the SPN was an incidental finding. Previous studies have shown that 70% of SPN cases are asymptomatic, while in 30% of cases it is detected incidentally.^[5,8]^ Once the tumor has grown enough to cause pressure symptoms to the adjacent organs, most patients will have corresponding clinical manifestations.^[8]^ This was evident in our case, where microscopic hematuria had persisted for 6 weeks and abdominal ultrasound revealed a large pancreatic mass, despite no complaints of discomfort and obvious self-evident symptoms.

According to relevant guidelines and diagnostic criteria,^[9,10]^ AMH or positive occult urine refers to the presence of red blood cells in the urine, which is generally found through laboratory examination of urine samples, and may be caused by renal internal diseases, trauma, drug factors, urinary tract infections, pyelonephritis, kidney stones and other factors. Urinary system tumors are not a common cause. In theory, it is difficult to link it to SPN. However, based on the case we reported, there is an association between AMH and SPN. Indeed, AMH caused by SPN is extremely rare and has not been reported in the literature. We speculate that their association may be due to the exogenous growth of this tumor involving the kidney. In other words, the kidney is being compressed by a huge mass (SPN). Obviously, this should be equivalent to a kidney injury, as postoperative (after relieving the pressure on the kidney) follow-up urine tests were normal and thus confirmed.

Epidemiology and pathogenesis: The incidence of SPN is low, accounting for approximately 0.17% to 2.7% of all pancreatic tumors.^[5]^ Law et al conducted a systematic review of bulk SPN cases, more than 87% of which were reported after 2000.^[11]^ The review showed that the number of cases reported in 2000 to 2012 increased 7-fold compared to 1961 to 1999, while men accounted for only 12.2%. The increase in the number of reported cases can be attributed to increased awareness and improved diagnostic capacity, rather than an actual increase in incidence. SPN usually occurs in the second or third decade of young adult female patients, with a male to female ratio of 1:11.^[5]^

There are 2 hypotheses about the pathogenesis of SPN. One hypothesis is that SPN does not belong to pancreatic exocrine cells or endocrine cells, but comes from the immature differentiation of pluripotent stem cells of the pancreas. The other is that SPN has a strong correlation with female germ cells, and the germ crista-ovarian primordium related cells migrate to the pancreatic primordium during histogenesis and gradually form tumors with the development of the pancreas.^[12,13]^ The spectrum of the disease, which tends to occur in young women, also suggests that the disease may be related to sex hormone receptors.^[14]^ In addition, mutations in p53 and K-ras genes common in pancreatic tumors do not appear to be significant in the occurrence of SPN, but chromosomal abnormalities and unbalanced karyotypic translocations may play a facilitating role in the development of this tumor.^[15,16]^

Histopathological and molecular characteristics: SPN is a low-grade malignant tumor. Approximately 60% of these are reported to be located in the body and tail of the pancreas, 35% in the head or uncinate process of the pancreas, and approximately 1% grow outside the pancreas, such as the adrenal glands, mesentery, ovary, retroperitoneum, and small intestine.^[11]^ The tumors are usually round and single, with well-defined boundaries.The section consisted of solid area, enlarged hemorrhagic necrotic area and pseudocystic cavity. Microscopic SPN growth morphology is very uniform,mainly composed of solid components, pseudopapillary or hemorrhagic pseudocyst structure.^[17]^ The solid area of the tumor is covered by poor adhesion epithelioid tumor cells, which degenerate and shed, while the remaining tumor cells form a pseudopapillary structure around the fibrous vascular bundle, hence the name SPN.It often presents a granulomatous reaction of cholesterol crystallization and foamy histiocytic cells. Its cytoplasm is eosinophilic or vacuolar, with PAS-D positive transparent spheroids. The nucleus is oval, concave, with scattered chromatin. Mitosis is uncommon and Ki-67 expression is low.

SPN lacks genetic alterations commonly seen in pancreatic ductal adenocarcinoma, such as KRAS, TP53, P16/ CDKN2A, and SMAD4. Its specific molecular marker is the point mutation of exon 3 of CTNNB1 gene, which is present in more than 90% of SPN and participates in the Wnt/ β-catenin signaling pathway.^[18,19]^ It has been found that transcription factors of various proteins upregulated in SPN, such as β-catenin, androgen receptor, lymphatic enhancer binding factor 1 (LEF1) and immunoglobulin heavy chain enhancer 3 (TFE3), play an important role in diagnosis. β-catenin is the most sensitive to the diagnosis of SPN, with a sensitivity of 100% and specificity of 87%, which is one of the histological diagnostic criteria of WHO.^[20]^

Clinical features and diagnosis: The clinical manifestations of SPN are usually nonspecific. Yao et al’s analysis of 2450 patients with SPN found that 51.6% had abdominal discomfort or pain, 40.2% had palpable abdominal mass, and 38.6% had no obvious symptoms, and the tumor was accidentally discovered through imaging.^[21]^ The mean tumor size of asymptomatic SPN patients was significantly smaller than that of symptomatic patients. Excessive tumor diameter can cause anorexia, dyspepsia, nausea and vomiting and other symptoms of digestive system pressure, and severe cases can be manifested as digestive tract obstruction, pancreatitis. Large tumors can also cause necrosis due to insufficient blood supply, leading to bleeding within the tumor, and even spontaneous rupture, causing hemorrhagic shock.

Since most patients with SPN have no specific symptoms, and most patients do not have abnormal tumor markers such as AFP, CEA, CA199, CA125, etc, and there are no diagnostic biomarkers for SPN, early, timely and accurate diagnosis of the disease remains a challenge.However, as more cases of SPN have been reported and knowledge of its pathology has increased, the understanding of its detection and diagnosis has also improved. Recently, biomarkers for SPN have been established to facilitate detection and diagnosis (such as LEF1,TFE3 etc).

For the imaging diagnosis of SPN, ultrasound can often detect SPN lesions, but qualitative diagnosis is difficult. The ultrasound findings of SPN are usually defined as a hypoechoic parenchymal mass containing a cystic area, a cystic mass,and semicircular calcification. CT is the mainstream radiological examination for SPN. The variation of CT attenuation value between solid, cystic, and hemorrhagic areas is obvious. Compared with CT, the main advantage of MRI is its better resolution on soft tissue. Consequently, there will be a significant advancement in the discrimination on the relationship between tumor and surrounding tissue.^[22]^ Enhanced CT and/or MRI are the imaging modalities of choice for evaluating resectable SPN and the presence or absence of metastasis, as well as for differential diagnosis. The imaging findings of SPN are often heterogeneous cystic solid mass with clear edges. Most of the solid components were located in the periphery, and most of the cystic components were located in the tumor. Some studies suggest that MRI is superior to CT in differentiating the internal structure of SPN, such as intracapsular hemorrhage, cystic degeneration, and tumor envelope.^[23]^ In recent years, texture analysis based on radiomics has been gradually applied to the diagnosis of pancreatic tumors. Li et al applied imaging omics technology to find that MRI texture analysis has a high sensitivity in distinguishing nonfunctional pancreatic neuroendocrine tumors from SPN, which may be better than the diagnostic effect of imaging physicians.^[24]^ PET/CT can also be used for the diagnosis of SPN, and its SUVmax value is mainly above 2.5, ranging from 2.4 to 44.8. The uptake of fluorodeoxyglucose (FDG) is usually positive, which may be related to the cell nature, proliferation index or histological malignancy of the tumor, but it is usually lower than that of pancreatic ductal adenocarcinoma and neuroendocrine tumor. It can be used to evaluate the difficulty of CT to distinguish benign and malignant pancreatic space and to determine the presence of distant metastasis.^[22]^

Treatment and prognosis: Based on the current understanding of the biological behavior of SPN, most guidelines assert that surgical intervention is the recommended approach for achieving comprehensive therapy of SPN, irrespective of the size of the tumor.^[25]^Surgery is the treatment of choice for SPN and over 95% of tumors can be resected (R0). Surgery can be curative even with distant metastases.^[26]^ Moreover, minimally invasive pancreatectomy may be a more effective option than open surgery.^[6]^ Common surgical procedures include pancreaticoduodenectomy, caudectomy, midpancreatic resection, and functional preservation pancreatic surgery, such as duodeno-sparing pancreaticotomy, tumor enucleation and other local surgical procedures. Nodal metastasis is very rare in SPN, and there is no consensus on whether to perform lymph node dissection in SPN. Lymph node dissection, which is routinely performed for pancreatic malignancies, is not appropriate for SPN.^[22]^ Kim et al reported lymph node recurrence of SPN and suggested complete resection of suspected metastatic lymph nodes, and pointed out that SPN larger than 5cm May benefit from lymph node dissection,^[27]^ but high-quality research evidence was still lacking.

However, although malignant SPN occurs in <15% of cases, excision may not always be appropriate due to the tendency to invade surrounding tissue and metastasize. For SPN that may be surgically removed, radical resection is recommended to minimize the possibility of recurrence. There is no universal evaluation criteria for the resection of SPN, and for resectable metastatic lesions, aggressive surgical resection is generally accepted.^[28]^ Radiofrequency ablation (RFA) is also an alternative option to consider when SPN is determined to be unresectable.^[22]^In addition to surgery, systemic chemotherapy or radiation therapy have been considered as potential treatments,^[29]^ but there is a lack of data to support them. Targeted therapy and hormone therapy have been reported for advanced unresectable SPN, but most have only been case reports.

SPN grows slowly, the degree of malignancy is low, and the overall prognosis is good. Recently, a study by Liu et al reported the largest cohort of patients with postoperative SPN from a single center and reported on the quality of life of these patients.^[30]^ Studies have shown that SPN is associated with excellent long-term survival, even in patients with metastasis, with most patients having a good quality of life after surgery. In another large cohort, SPN was associated with good long-term survival after surgery. For malignant SPN, incomplete tumor envelope is an independent risk factor.^[31]^ Law et al systematically evaluated 1952 patients with SPN, 95.6% of them survived without tumor, 4.4% of them had tumor recurrence, and the median time of tumor recurrence was about 50.5 months. Non-R0 resection, large tumor size, young patient age, tumor rupture, and male patients were risk factors for postoperative recurrence.^[11]^ More recently, Liu et al analyzed long-term quality of life in patients with SPN who underwent radical surgery.^[30]^ Most patients do well on quality of life measures such as relationships, marriage and family, but still approximately 20% of patients experience a decline in quality of life 3 to 6 years after surgery. Therefore, although most patients with SPN have a good prognosis, due to the relative instability of their biological behavior, annual lifelong follow-up is recommended even after radical surgery is completed.

## 4. Limitations

This study reports a case of a patient with SPN presenting with AMH as the initial manifestations. Based on this case, we hypothesize that SPN may be one of the causes of AMH. However, there are some limitations in our study. The main limitation is that the hypothesis was proposed based on a single case, and future studies need to verify the causal relationship through multicenter cohort studies. Other limitations include the lack of sufficient data support between SPN and AMH, and the relatively short follow-up time.

## 5. Conclusions

Frantz tumor, or SPN, is a rare pancreatic tumor that occurs in young women, and its etiology and pathogenesis remain unclear. Most patients are usually asymptomatic or atypical, and are often found during outpatient visits for other reasons or may be incidentally found during imaging tests. Suspicion and proper investigation are essential to diagnose this entity in a timely manner. Surgical resection remains the preferred treatment for radical treatment and long-term prognosis. To increase the early diagnosis rate of SPN, explore the comprehensive treatment of recurrent and metastatic tumors, and improve the long-term postoperative quality of life of patients are the focus of future research.

## Acknowledgments

The authors thank the faculty of the Science and Education Department of Hebei Cangzhou Hospital of Integrated Traditional Chinese Medicine and Western Medicine for their continued help and attention in the study.

## Author contributions

**Conceptualization:** Haixia Ren.

**Data curation:** Haixia Ren, Chongxi Ren, Sheng Li.

**Formal analysis:** Haixia Ren, Chongxi Ren, Sheng Li.

**Investigation:** Haixia Ren, Chongxi Ren, Sheng Li.

**Methodology:** Haixia Ren, Chongxi Ren, Sheng Li.

**Resources:** Chongxi Ren, Sheng Li.

**Supervision:** Haixia Ren, Chongxi Ren, Sheng Li.

**Validation:** Haixia Ren, Chongxi Ren, Sheng Li.

**Writing – original draft:** Haixia Ren, Chongxi Ren, Sheng Li.

**Writing – review & editing:** Haixia Ren, Chongxi Ren, Sheng Li.

## References

[1] Dinarvand P Wang WL Roy-Chowdhuri S. Utility of SOX11 for the diagnosis of solid pseudopapillary neoplasm of the pancreas on cytological preparations. Cytopathology. 2022;33:216–21. 34816516 10.1111/cyt.13080 PMC8813899

[2] Tostes FT de Carvalho PFDC Araújo RLC. Clinical course, genetic, and immunohistochemical characterization of solid pseudopapillary tumor of the pancreas (Frantz Tumors) in a Brazilian Cohort. Genes (Basel). 2022;13:1809. 36292694 10.3390/genes13101809 PMC9601385

[3] Hu R Gui R Nie X Duan H. Case report: clinical analysis and literature review of five cases of metastatic solid pseudopapillary tumor of the pancreas. Front Oncol. 2024;14:1386987. 39450257 10.3389/fonc.2024.1386987 PMC11499064

[4] Torres OJM Rezende MB Waechter FL. Pancreatoduodenectomy for solid pseudopapillary tumor of the pancreas: a multi-institution study. Arq Bras Cir Dig. 2019;32:e1442. 31460602 10.1590/0102-672020190001e1442 PMC6713058

[5] Chen CC Feng TY Jan HC Chou SJ Chen TH Wang SC. Rare case of solid pseudopapillary neoplasm of the pancreas with liver and splenic metastasis in a 19-year-old girl. Int J Surg Case Rep. 2024;120:109867. 38870658 10.1016/j.ijscr.2024.109867 PMC11258626

[6] Dalili A Aliakbarian M Karimi-Shahri M Samadi A Raji S. Solid Pseudopapillary neoplasms are rare, indolent pancreatic tumors in young women. Case Rep Surg. 2020;2020:6694904. 33299632 10.1155/2020/6694904 PMC7704190

[7] Sohrabi C Mathew G Maria N Kerwan A Franchi T Agha RA; Collaborators. The SCARE 2023 guideline: updating consensus Surgical CAse REport (SCARE) guidelines. Int J Surg. 2023;109:1136–40. 37013953 10.1097/JS9.0000000000000373 PMC10389401

[8] AlQattan AS Alshaqaq HM Al Abdrabalnabi AA Alnamlah M Alanazi AA Alqahtani MS. Huge solid pseudopapillary tumor of the pancreas ‘Frantz tumor’: a case report. J Gastrointest Oncol. 2020;11:1098–104. 33209501 10.21037/jgo-20-180 PMC7657821

[9] Whiteside JL Yuen HTH. Asymptomatic microscopic hematuria in women. Curr Opin Obstet Gynecol. 2019;31:471–6. 31592827 10.1097/GCO.0000000000000573

[10] Linder BJ Bass EJ Mostafid H Boorjian SA. Guideline of guidelines: asymptomatic microscopic haematuria. BJU Int. 2018;121:176–83. 28921833 10.1111/bju.14016

[11] Law JK Ahmed A Singh VK. A systematic review of solid-pseudopapillary neoplasms: are these rare lesions? Pancreas. 2014;43:331–7. 24622060 10.1097/MPA.0000000000000061 PMC4888067

[12] Stark A Donahue TR Reber HA Hines OJ. Pancreatic cyst disease: a review. JAMA. 2016;315:1882–93. 27139061 10.1001/jama.2016.4690

[13] Naar L Spanomichou DA Mastoraki A Smyrniotis V Arkadopoulos N. Solid pseudopapillary neoplasms of the pancreas: a surgical and genetic enigma. World J Surg. 2017;41:1871–81. 28251269 10.1007/s00268-017-3921-y

[14] Lee WY Tzeng CC Chen RM Tsao CJ Tseng JY Jin YT. Papillary cystic tumors of the pancreas: assessment of malignant potential by analysis of progesterone receptor, flow cytometry, and ras oncogene mutation. Anticancer Res. 1997;17:2587–91. 9252685

[15] Pettinato G Di Vizio D Manivel JC Pambuccian SE Somma P Insabato L. Solid-pseudopapillary tumor of the pancreas: a neoplasm with distinct and highly characteristic cytological features. Diagn Cytopathol. 2002;27:325–34. 12451561 10.1002/dc.10189

[16] Maitra A Weinberg AG Schneider N Patterson K. Detection of t(11;22)(q24;q12) translocation and EWS-FLI-1 fusion transcript in a case of solid pseudopapillary tumor of the pancreas. Pediatr Dev Pathol. 2000;3:603–5. 11000339 10.1007/s100240010119

[17] Kosmahl M Seada LS Jänig U Harms D Klöppel G. Solid-pseudopapillary tumor of the pancreas: its origin revisited. Virchows Archiv. 2000;436:473–80. 10881741 10.1007/s004280050475

[18] Abraham SC Klimstra DS Wilentz RE. Solid-pseudopapillary tumors of the pancreas are genetically distinct from pancreatic ductal adenocarcinomas and almost always harbor beta-catenin mutations. Am J Pathol. 2002;160:1361–9. 11943721 10.1016/s0002-9440(10)62563-1 PMC1867216

[19] Tanaka Y Kato K Notohara K. Frequent beta-catenin mutation and cytoplasmic/nuclear accumulation in pancreatic solid-pseudopapillary neoplasm. Cancer Res. 2001;61:8401–4. 11731417

[20] Harrison G Hemmerich A Guy C. Overexpression of SOX11 and TFE3 in solid-pseudopapillary neoplasms of the pancreas. Am J Clin Pathol. 2017;149:67–75. 29272888 10.1093/ajcp/aqx142

[21] Yao J Song H. A review of clinicopathological characteristics and treatment of solid pseudopapillary tumor of the pancreas with 2450 cases in Chinese population. Biomed Res Int. 2020;2020:2829647. 32685461 10.1155/2020/2829647 PMC7352122

[22] Lu X Chen H Zhang T. Solid pseudopapillary neoplasm (SPN) of the pancreas: current understanding on its malignant potential and management. Discover Oncol. 2024;15:77. 10.1007/s12672-024-00905-5 PMC10948659 38498246

[23] Li DL Li HS Xu YK Wang QS Chen RY Zhou F. Solid pseudopapillary tumor of the pancreas: clinical features and imaging findings. Clin Imaging. 2018;48:113–21. 29073488 10.1016/j.clinimag.2017.10.006

[24] Li X Zhu H Qian X Chen N Lin X. MRI texture analysis for differentiating nonfunctional pancreatic neuroendocrine neoplasms from solid pseudopapillary neoplasms of the pancreas. Acad Radiol. 2020;27:815–23. 31444110 10.1016/j.acra.2019.07.012

[25] European Study Group on Cystic Tumours of the Pancreas. European evidence-based guidelines on pancreatic cystic neoplasms. Gut. 2018;67:789–804. 29574408 10.1136/gutjnl-2018-316027 PMC5890653

[26] Silano F de Melo Amaral RB Santana RC Neves VC Ardengh JC do Amaral PCG. Yield of surgery in solid pseudopapillary neoplasms of the pancreas: a case series and literature review. World J Gastrointest Oncol. 2021;13:589–99. 34163575 10.4251/wjgo.v13.i6.589 PMC8204350

[27] Kim MJ Choi DW Choi SH Heo JS Sung JY. Surgical treatment of solid pseudopapillary neoplasms of the pancreas and risk factors for malignancy. Br J Surg. 2014;101:1266–71. 25052300 10.1002/bjs.9577

[28] Zhang C Liu F Chang H. Less aggressive surgical procedure for treatment of solid pseudopapillary tumor: limited experience from a single institute. PLoS One. 2015;10:e0143452. 26599966 10.1371/journal.pone.0143452 PMC4658154

[29] Maffuz A Silva JA Torres-Vargas S. Preoperative gemcitabine for unresectable, solid pseudopapillary tumour of the pancreas. Lancet Oncol. 2005;6:185–6. 15737835 10.1016/S1470-2045(05)01770-5

[30] Liu Q Dai M Guo J. Long-term survival, quality of life, and molecular features of the patients with solid pseudopapillary neoplasm of the pancreas: a retrospective study of 454 cases. Ann Surg. 2023;278:1009–17. 37036095 10.1097/SLA.0000000000005842

[31] Fu C Li X Wang Y. Solid pseudopapillary neoplasm of the pancreas: a retrospective study of 195 cases. Front Oncol. 2024;14:1349282. 38469229 10.3389/fonc.2024.1349282 PMC10925641

